# Illinois

**Published:** 1897-01

**Authors:** 


*Illinois.* According to State Veterinarian Trumbower, Illinois cattle are less troubled with disease than at any time the past year, but swine-plague is prevalent in the northern part of the State. During the year's post-mortem inspection at the stock-yards only 78 cases of tuberculosis were found and 21 cases of cancer. Carcasses of 802 cattle were passed on post-mortem examination and 1643 condemned and ordered tanked.

